# Enhancing balance and auditory function in bilateral vestibulopathy through otolithic vestibular stimulation: insights from a pilot study on cochlea-vestibular implant efficacy

**DOI:** 10.3389/fneur.2025.1520554

**Published:** 2025-01-30

**Authors:** Ángel Ramos-de-Miguel, Morgana Sluydts, Juan Carlos Falcón, Raquel Manrique-Huarte, Isaura Rodriguez, Andrzej Zarowski, Maurizio Barbara, Manuel Manrique, Silvia Borkoski, Joan Lorente, Marc Leblanc, Antonin Rambault, Erika Van Baelen, Carl Van Himbeeck, Alicia Huarte, Ángel Ramos Macías

**Affiliations:** ^1^Unit of Hearing Loss, Department of Otorhinolaryngology, Head and Neck, Complejo Hospitalario Universitario Insular Materno Infantil, Las Palmas of Gran Canaria, Spain; ^2^Universidad Las Palmas de Gran Canaria, Las Palmas of Gran Canaria, Spain; ^3^European Institute for Otorhinolaryngology, GZA Hospitals Antwerp, Wilrijk, Belgium; ^4^Department of Otorhinolaryngology, Clinica Universidad de Navarra, Pamplona, Spain; ^5^Department of NESMOS, ENT Clinic, Sapienza University, Rome, Italy; ^6^Cochlear Technology Centre, Mechelen, Belgium

**Keywords:** bilateral vestibular dysfunction, otolithic implants, cochleovestibular implant, dynamic gait index, balance improvement, vestibular rehabilitation

## Abstract

**Introduction:**

This study evaluates cochleovestibular implants (CVI) for improving auditory and vestibular function in patients with bilateral vestibulopathy and severe-to-profound hearing loss. CVI uniquely combines auditory and vestibular stimulation, offering a potential solution for dual impairments in hearing and balance.

**Methods:**

Ten patients underwent CVI implantation. Auditory function was assessed with pure-tone audiometry (PTA) and speech recognition at baseline, 3, and 9 months post-implantation. Vestibular function was measured using the Dynamic Gait Index (DGI), Sensory Organization Test (SOT), posturography, and the Dizziness Handicap Inventory (DHI). Placebo-controlled tests verified cochleovestibular stimulation specificity.

**Results:**

Auditory outcomes showed PTA improvements from 78 dB HL preoperatively to 34 dB HL at 3 months and 36 dB HL at 9 months, alongside speech recognition gains. Vestibular improvements included significant DGI (*p* < 0.05) and SOT score increases (33% to 68%, *p* < 0.05). DHI scores reflected reduced dizziness-related disability. Residual balance gains after device deactivation suggest neural adaptation, and placebo tests confirmed cochleovestibular stimulation specificity.

**Discussion:**

CVI effectively restores auditory and vestibular function, with improvements in hearing, balance, and quality of life. Neural plasticity likely supports long-term benefits. Future research should refine device design and stimulation protocols to enhance outcomes further.

## 1 Introduction

Bilateral vestibulopathy (BVP) significantly impairs patients’ quality of life by causing chronic instability, vertigo while standing or walking, and oscillopsia, particularly in low-light conditions (1). This condition, with a prevalence of 16–28 cases per 100,000 people, disproportionately affects older adults, leading to severe consequences such as increased risk of falls and loss of independence (2, 3). Despite advances in rehabilitation therapies, many patients fail to achieve satisfactory improvements in balance and spatial orientation (4, 5).

Vestibular implants (VIs) have emerged as a promising therapeutic option for restoring vestibular function in patients with BVP. These devices aim to restore equilibrium by providing electrical stimulation to the vestibular organs, potentially improving both balance and quality of life. While VIs are still in the developmental stage, early studies suggest their effectiveness in compensating for lost vestibular function, offering hope for patients unresponsive to conventional therapies. Currently there are two main approaches for vestibular implants: semicircular canal (SCC) implants that focus on gaze stabilization and otolithic implants that prioritize postural stability (6).

Among the key structures of the vestibular system, the otoliths, that houses the utricle and saccule of the inner ear, play a fundamental role in detecting gravity and linear accelerations. These sensory organs contain hair cells covered by an otolithic membrane, which is embedded with calcium carbonate crystals known as otoconia. This system provides crucial information to the brain for maintaining balance and spatial orientation. The stimulation of otoliths is essential for restoring vestibular function in patients with BVP, as the absence of such stimulation—common in bilateral vestibulopathy—results in a failure to detect gravitational forces, leading to severe instability and an increased risk of falls (7).

First attempts in vestibular implants have been focussed on semicircular canal stimulators aiming to restore the function of the semicircular canals, which are primarily responsible for detecting angular accelerations and stabilizing gaze (6, 8–10).

Two main prototypes for VIs have been developed to stimulate the semicircular canals. The Marburg-Los Angeles prototype, designed by Golub et al. (11) and Phillips et al. (12), is based on a modified cochlear implant adapted for afferent stimulation of the semicircular canals. This device includes two extracochlear reference electrodes (plate and ball) and three electrode arrays designed to stimulate the canals, each containing three electrode contacts. Initially tested in animal models, this vestibular pacemaker was then trialed in humans, where the electrodes were implanted in the perilymphatic space of the preferred semicircular canal to stimulate its afferent pathways. The aim of this system was to counteract vestibular symptoms during attacks in patients with disabling Ménière’s disease. However, only one patient reported a mild Ménière’s episode lasting about an hour during the trial.

The Maastricht-Geneva prototype, studied by Guyot et al. (8–10), also uses a modified cochlear implant, incorporating one to three vestibular electrodes for semicircular canal stimulation, alongside an intracochlear electrode array.

SCC implants offer several key benefits for patients with bilateral vestibulopathy. One of their primary advantages is improving gaze stability. The main function of the SCCs is to stabilize vision during head movements by restoring the vestibulo-ocular reflex (VOR), allowing patients to maintain clear vision even while in motion. Additionally, SCC stimulation helps reduce oscillopsia, a common symptom in vestibular dysfunction where the environment appears to move. By enhancing VOR function, SCC implants can alleviate this disorienting sensation, leading to an improved quality of life for affected individuals (13–15).

Electrical stimulation of the semicircular canals can induce a temporary tonic asymmetry, to which patients typically adapt. This side effect may present as vertigo, nausea, and nystagmus, but these symptoms are usually managed through a period of adjustment to the stimulation. SCC implants demonstrate effectiveness in improving gaze stability and reducing oscillopsia (13, 14).

Clinical observations and studies suggest that the otolithic system has certain advantages over the semicircular canals in maintaining equilibrium and detecting gravity (7).

The otolithic system is notably robust, with unilateral dysfunctions often going undetected in clinical assessments. For instance, in cases of vestibular neuritis affecting the superior vestibular nerve (which innervates the utricle and semicircular canals), symptoms like rotational vertigo and horizontal nystagmus are more prominent, while otolithic dysfunction may only become apparent in severe conditions like Tumarkin’s syndrome. Additionally, the otolithic organs can inhibit signals from the semicircular canals, contributing to enhanced overall stability. Unlike the semicircular canals, the otolithic membrane is not based on a spring-damped system, allowing for continuous detection of the gravitational vector.

Given the otoliths’ crucial role in maintaining balance, their stimulation through vestibular implants is vital for improving a patient’s stability and quality of life. Otolith implants, which primarily stimulate the inferior branch of the vestibular nerve (mostly innervating the saccule), aim to restore lost vestibular function in patients with bilateral vestibular loss, offering significant improvements in their daily functioning.

One of the core advantages of otolithic stimulation is its ability to restore gravity detection (7). Healthy otoliths continuously sense gravity, providing the brain with essential information about head orientation. When this function is compromised, it leads to imbalance and an increased risk of falls. Electrical stimulation of the otoliths can recreate this gravitational reference, improving the patient’s perception of orientation and overall posture.

Another benefit of otolith implants is the potential for unilateral stimulation. Unlike semicircular canal implants, which often require bilateral intervention, otolithic stimulation on one side may be sufficient to restore vestibular function. This is due to the otolithic system’s robust nature, which does not rely on the bilateral agonist–antagonist mechanism seen in the semicircular canals.

This article aims to evaluate, for the first time, the safety and efficacy on otolithic stimulation for vestibular implants. The current research is focusing on three critical aspects: First the impact of the otolith stimulation on hearing. Second, evaluate the vestibular benefit. Third, analyzed the chronic long-term effect of vestibular stimulation.

## 2 Methodology

### 2.1 Participants

The clinical trial requires participants to meet the Bárány Society’s criteria for BVP (16), including a VOR gain of less than 0.6 in both ears, and to have severe to profound bilateral hearing loss qualifying for a cochlear implant. Candidates must be 18 years or older, have experienced vestibular symptoms for over a year without improvement, and have no expectation of recovery. Suitable inner ear anatomy, confirmed by imaging, is also required, along with the absence or difficulty in reproducing cVEMP and oVEMP in the targeted ear.

Exclusion criteria include inability to provide informed consent, normal vestibular function, unilateral or compensated vestibular loss, and central or cerebellar dysfunction (e.g., CANVAS syndrome). Patients with Persistent Postural-Perceptual Dizziness (PPPD), mild to moderate hearing loss, inner ear anomalies preventing full electrode insertion, or retrocochlear/central hearing dysfunction are also excluded. Other exclusion criteria are medical contraindications for surgery, psychiatric conditions, unrealistic expectations, substance abuse, vestibular suppressants, and disorders like orthostatic tremor, oculomotor dysfunction, or peripheral neuropathies. Conditions such as Parkinson’s disease, atypical parkinsonian syndromes, multisystem atrophy, and other central gait disorders also disqualify candidates.

### 2.2 Study protocol

**Figure 1:**
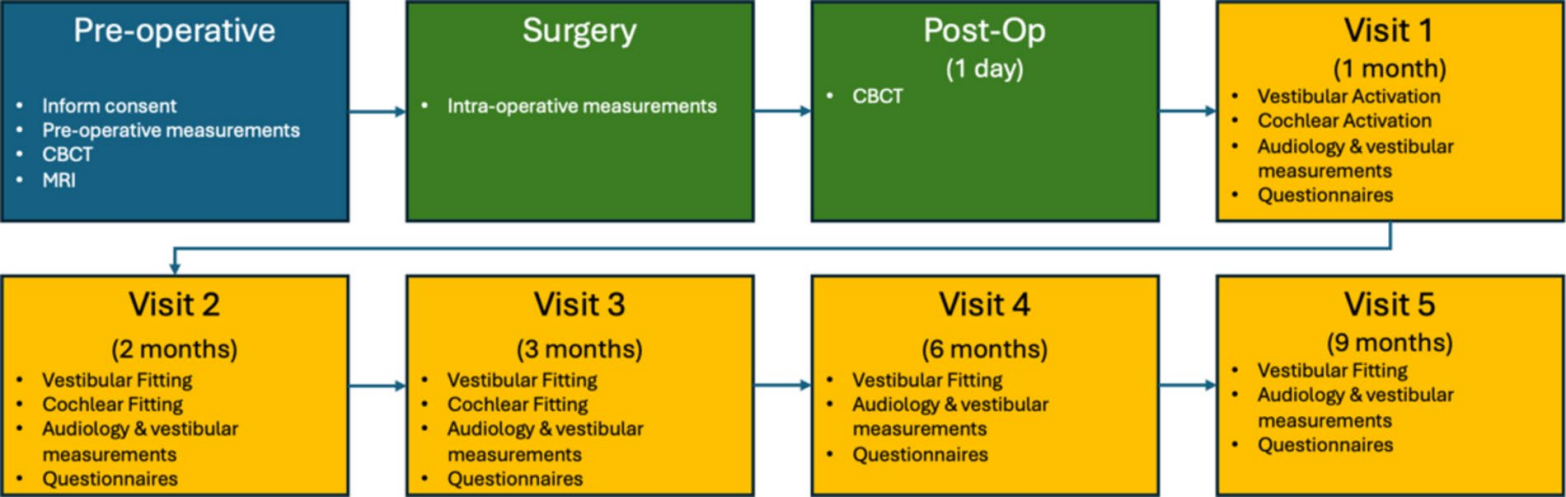
The road map was provided to the participants to visualize the extensiveness of the study.

A prospective, within-subject repeated-measures study with a 9-month follow-up was conducted. The protocol included auditory and vestibular assessments before and after implantation across multiple visits. Figure 1 illustrates the study program roadmap.

Preoperative tests were conducted within 2 months prior to implantation, while postoperative tests were performed during scheduled follow-up visits at one, 2, 3, 6, and 9-months post-implantation. The tests were conducted under two conditions: with the implant deactivated (OFF), meaning no saccular or cochlear stimulation, and with the implant activated (ON), where saccular and cochlear stimulation were applied simultaneously.

#### 2.2.1 Tests conducted

In all cases, the following tests were conducted to present the study’s findings: demographic characteristics, hearing assessments (including Pure Tone Audiometry (PTA) (250, 500, 1,000, 2000 and 4,000 Hz) and a speech test in silence), and vestibular evaluations [Dynamic Gait Index (DGI), posturography, Video Head Impulse Test (VHIT) and the Timed Up and Go (TUG) test]. Additionally, implant electrical tests were performed by using Trans-Impedance Matrix (TIM).

Quality of life was evaluated through questionnaires, including the Dizziness Handicap Inventory (DHI). Imaging studies, including pre-operative Magnetic Resonance Imaging (MRI), pre-operative Cone Beam Computed Tomography (CBCT), and post-operative CBCT, were also conducted.

#### 2.2.2 Cochleo-vestibular implant (CVI)

**Figure 2:**
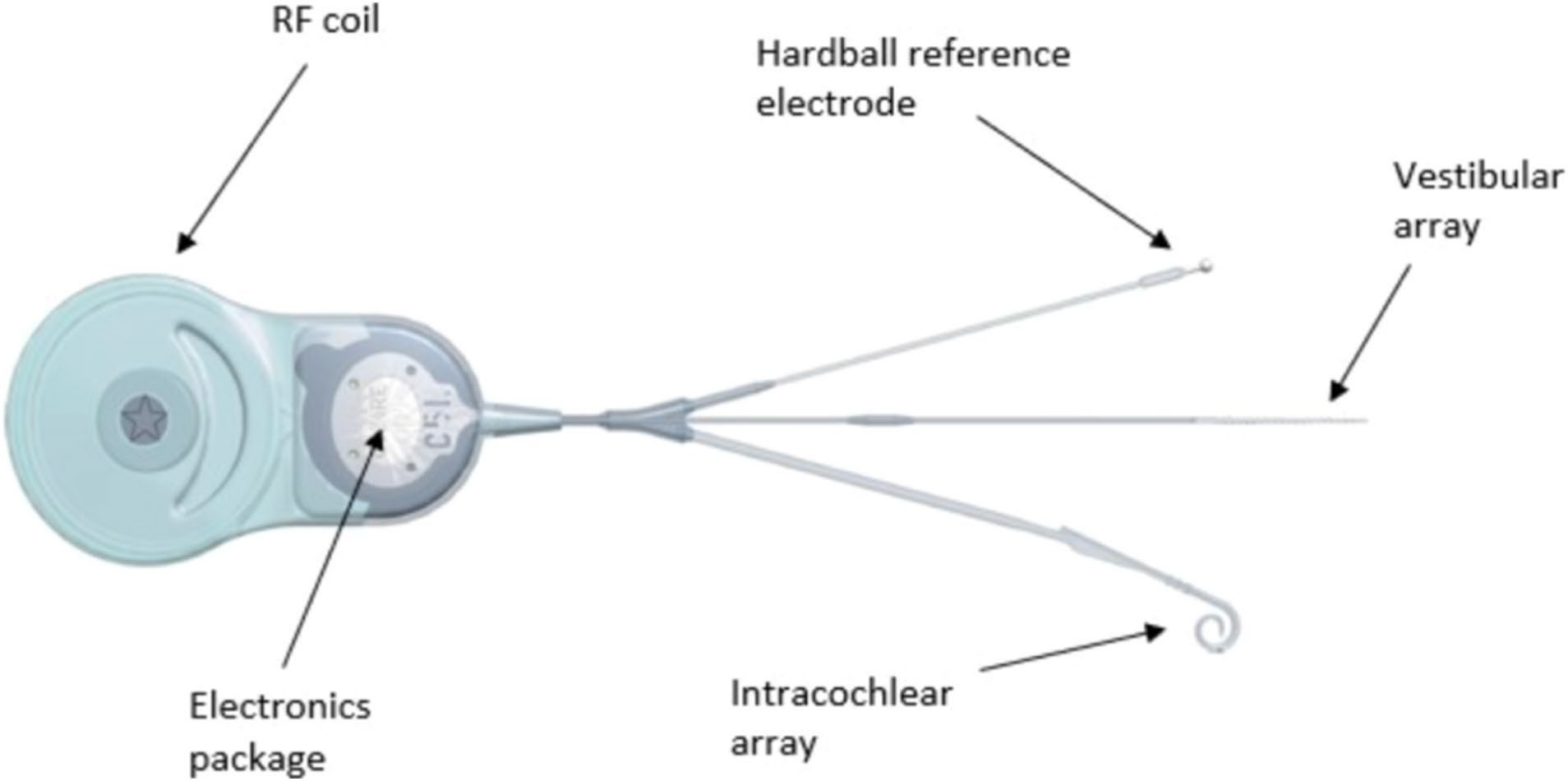
Internal part of the cochleovestibular implant system with coil, intra-cochlear electrode array (19 electrode contacts), vestibular array (three electrode contacts) and reference ball electrode. RF, radio frequency.

The Bionic\Vest implant system consists of two main components: an internal part and an external part. The internal components includes a coil, an intracochlear electrode array featuring 19 contacts in a CI512 electrode array shape, a vestibular array with three full-band electrode contacts with a 0.2 mm spacing between electrodes, and a reference ball electrode (Figure 2). The external part is identical to the commercially available Nucleus® 6 or 7 sound processor produced by Cochlear® in New South Wales, Australia.

For the placement of the implant, the stimulating contacts of the vestibular electrode array are positioned near the inferior vestibular nerve in the saccular region through a 0.5 mm stapedotomy. The cochlear electrode array is inserted into the cochlea via an incision in the anterior part of the round window membrane, or preferably through the classical cochleostomy. The reference electrode is placed traditionally, underneath the periosteum, directed toward the zygomatic arch.

Regarding stimulation parameters, biphasic pulse trains with balanced charge are generated. The pulse width is set at 25 μs, and the stimulation frequency is 900 pulses per second. Monopolar stimulation is used, with the ball and plate electrode serving as the reference, MP1 + 2.

In terms of stimulation modulation, cochlear stimulation involves the modulation of stimuli in amplitude between the threshold level (T) and the comfort level (C), thereby defining the dynamic range. Meanwhile, otolith stimulation is maintained at a constant current level.

### 2.3 Statistical analysis

Whenever possible (i.e., sample size large enough), statistical analyses were performed on the group data (PTA, TUG, DGI) with IBM® SPSS® Statistics, version 25 (IBM, Armonk, NY, USA). A significance level of 0.05 was used.

For the PTA and TUG data, the non-parametric Friedman Related Samples Two-Way Repeated Analysis of Variance by ranks was used because of the small sample size (*n* = 10). Pairwise *post hoc* analyses were performed when applicable (Wilcoxon Signed Ranks Test). The Bonferroni adjustment for multiple comparisons was applied, leading to adjusted probability (p) values. In other words, the obtained *p* values were multiplied by the number of comparisons carried out, leading to the adjusted p values, which were reported below.

For the DGI, a linear mixed model (LMM) analysis could be performed as well. The following terms were entered in the model: study visit (“visit”), CVI ON or OFF (“condition”), interaction effect “condition*visit.” The dependent variable was the total DGI score. The interaction effect term was removed from the model as it showed no significant effect. Doing so, however did not reduce the maximum likelihood (−2 Log Likelihood). Therefore, the first model was used for further interpretation of the data. The multiple *post-hoc* pairwise comparisons required Bonferroni adjustment of the *p*-values, which were reported.

### 2.4 Ethical considerations

The study received ethical approval from the competent authorities in all participating countries (Spain, Belgium, Italy) and their respective local ethics committees. Written informed consent was obtained from all participants.

The current study was performed in accordance with the 1964 Helsinki Declaration and its later amendments or comparable ethical standards, where applicable. All patients provided written informed consent before participating. All the procedures involving human participants were in accordance with the ethical standards of our institutional research committees.

## 3 Results

The study initially included 11 participants diagnosed with BVP and severe to profound bilateral hearing loss. Unfortunately, one participant was unable to complete the study due to complications from COVID-19, which ultimately resulted in his passing away before finishing the study.

The study’s demographics (*n* = 11) indicate a mean age at inclusion of 54.9 years (SD: 14.6) and a median age of 58 years, with an interquartile range (IQR) from 49 to 66.5 years. The participant group consisted of 27% females (3 out of 11) and 73% males (8 out of 11). Tinnitus was reported in the right ear by 45% of participants (5 out of 11) and in the left ear by 36% (4 out of 11). Initially, 64% (6 out of 11) used a hearing aid in the right ear, with 18% (2 out of 11) continuing its use after VI. For the left ear, 45% (5 out of 11) used a hearing aid at inclusion, with 27% (3 out of 11) maintaining its use following VI.

### 3.1 Electrical evaluation

**Figure 3:**
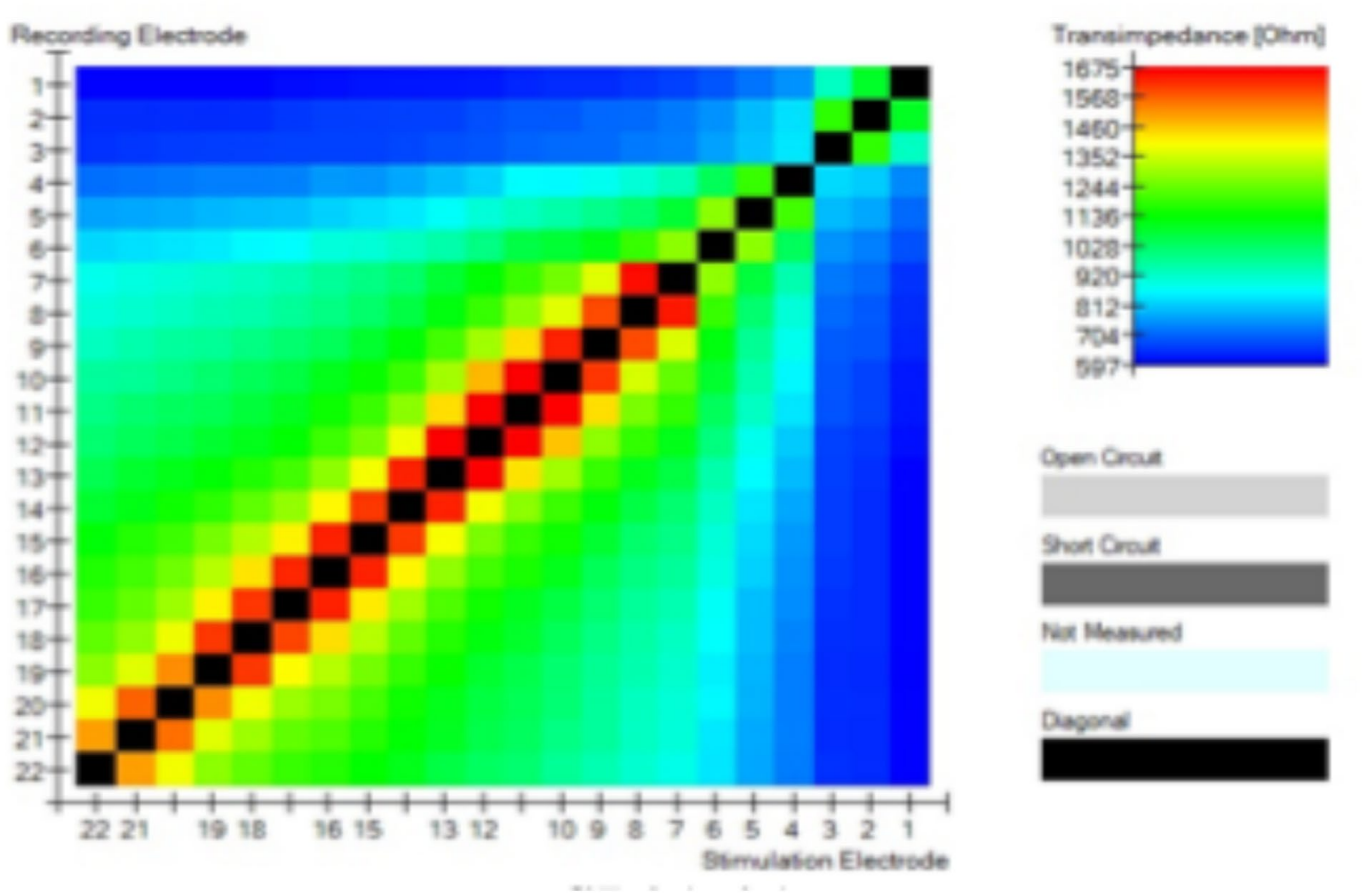
Trans-impedance recordings with the CI24RE (VEST). All figures show two different spaces: one for the vestibular electrode contacts (E1, E2, and E3) and one for the cochlear electrode contacts (E4–E22). No current flow could be observed between the cochlea and the vestibular space.

The aim is to analyze the effect of electrical vestibular stimulation on the cochlea, and of electrical cochlear stimulation on the vestibule. In all cases, the TIM results show that there is a discontinuity between the cochlear and vestibular spaces. This is shown in Figure 3, there is a clear change in the transimpedance values in the area where the stimulus is produced in another place where it is recorded. For example, when we stimulate electrode 1 (vestibular) and record at electrode 4 (cochlear) the transimpedance value is very low.

### 3.2 Hearing evaluation

#### 3.2.1 Pure tone audiometry

The mean pre-operative PTA [mean = 78 dB HL; standard deviation (SD) = 12 dB HL] improved statistically significant during the aided condition (CVI ON) when compared to the 3 months (*p* < 0.05; mean = 34 dB HL; SD = 16 dB HL) and 9 months (*p* < 0.05; mean = 36 dB HL; SD = 18 dB HL) postoperative visits.

**Figure 4:**
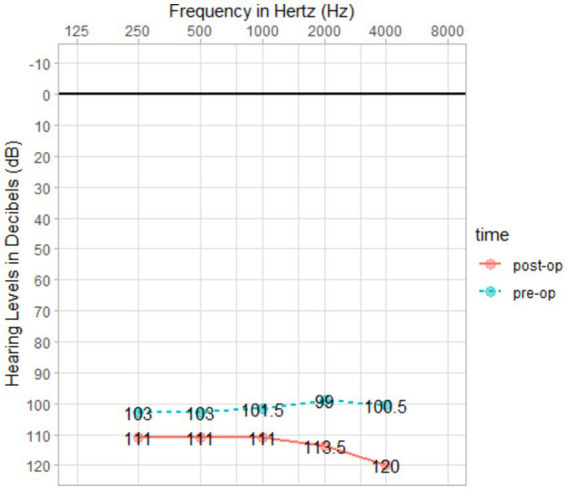
Pre- and post-operative PTA.

No unexpected changes were observed regarding the hearing in the implanted ears during the unaided ear-specific evaluations. After the surgery, the little residual hearing that was left in some patients was completely lost because of the insertion of the CI512 electrode array, which was an anticipated consequence of the surgical procedure (Figure 4).

#### 3.2.2 Word test in silence

**Figure 5:**
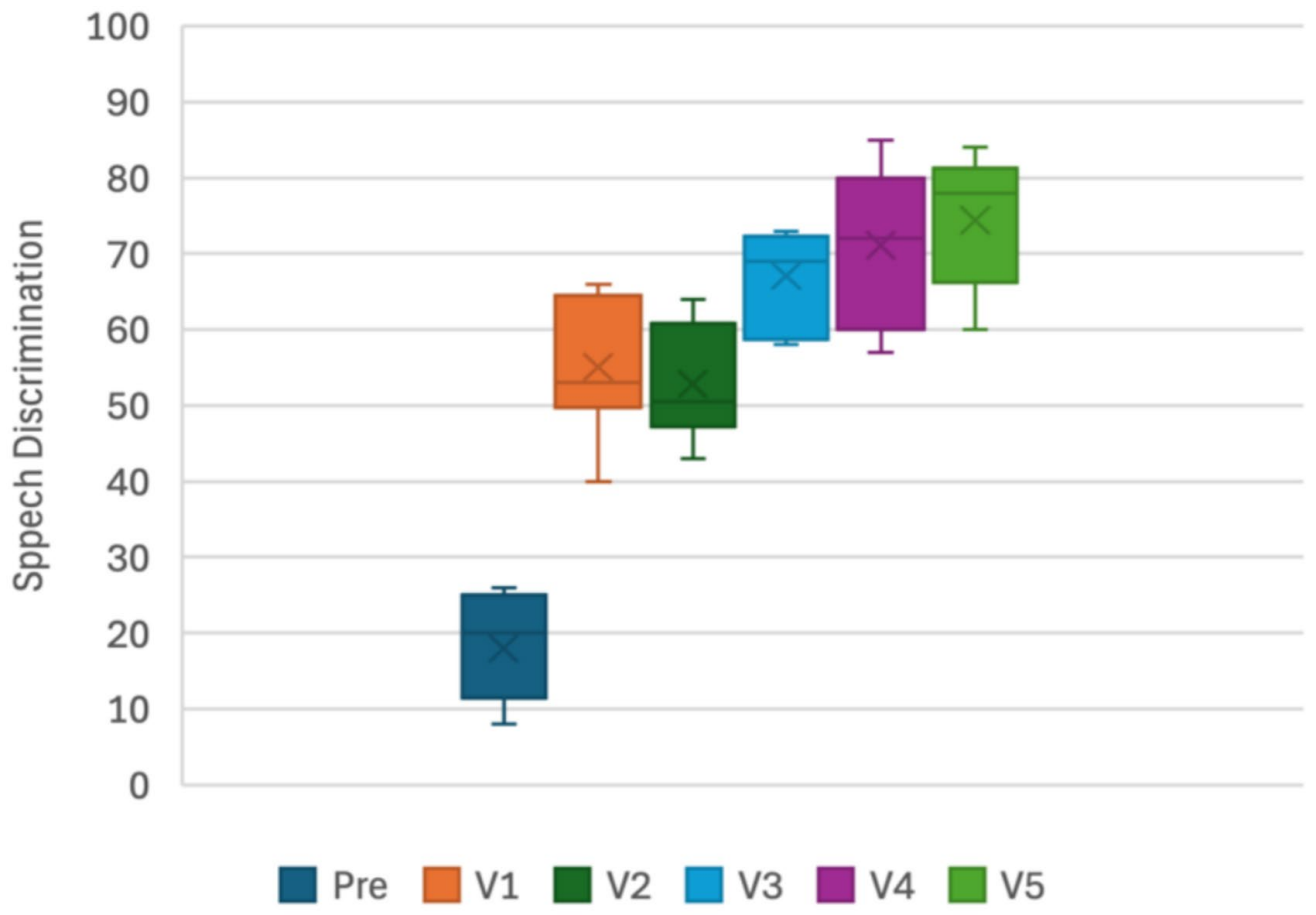
Speech recognition in silence.

Recorded materials were presented at 65-dB sound pressure level in the sound field with the subject seated 1 m from the speaker facing a 0-degree azimuth. A calibrated compact disc was used rather than a live voice. The variable to be recorded for speech in silence is the “% correct words” at 65-dB sound pressure level for 2 lists of 25 words. The materials used in this speech test can include monosyllabic or disyllabic words lists in the patients’ native language (Figure 5).

### 3.3 Vestibular evaluation

#### 3.3.1 Dynamic gait index

**Figure 6:**
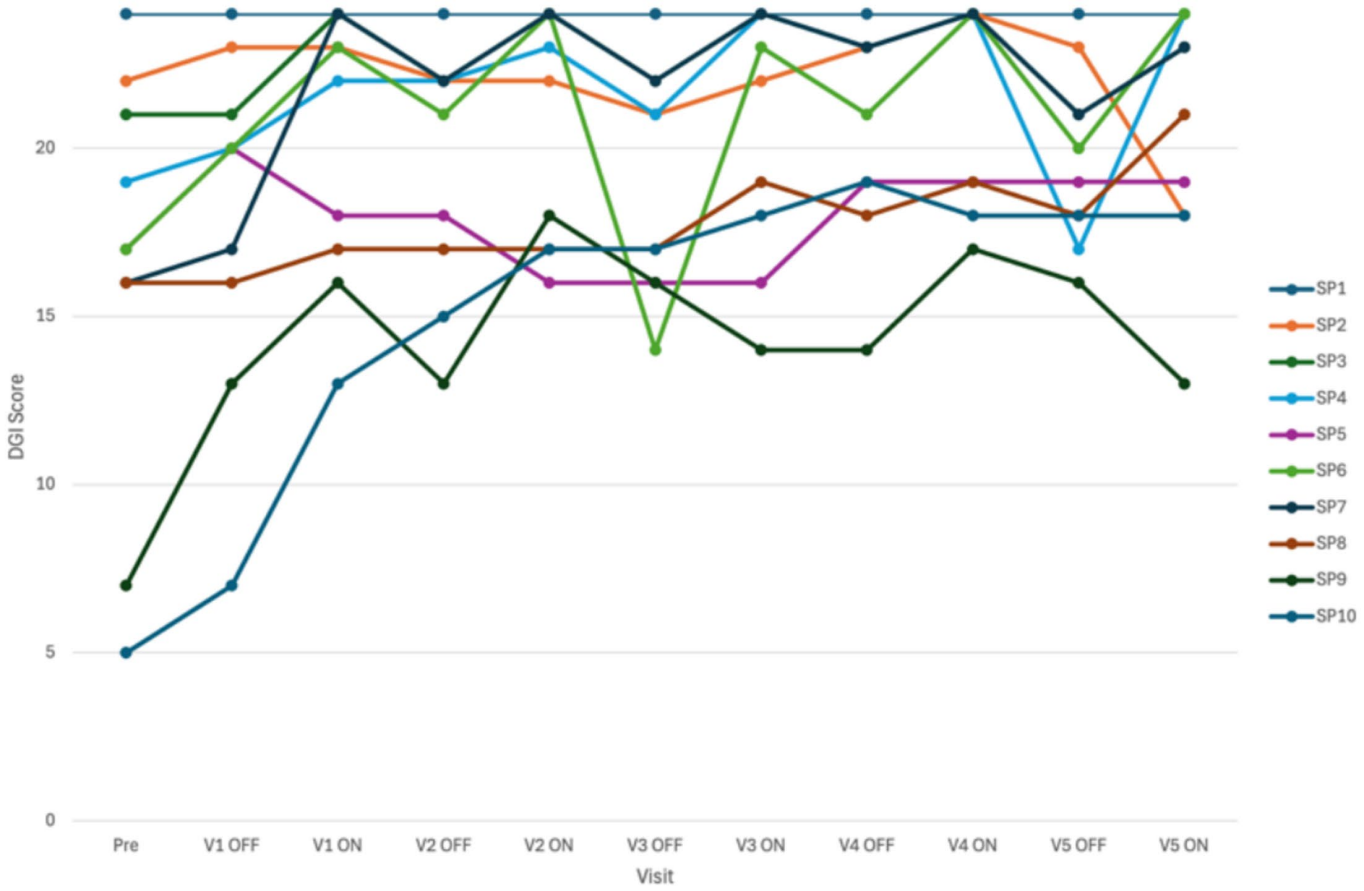
Difference in total score on the dynamic gait index (DGI) between study visits and condition of the cochleovestibular implant (CVI; ON or OFF) (*n* = 10).

A normal range was observed after the second visit. All patients improved (*p* < 0.05) and 9 cases were found within the normal range (>20–22). In three cases no improvement was found, likely because of a ceiling effect as they had normal DGI score before surgery (Figure 6).

The results of the LMM showed that there was a significant effect of “visit” [*F*_(5, 41)_ = 6,651; *p* < 0.001] and “condition” [*F*_(1, 42)_ = 22,785; *p* < 0.001]. No significant interaction effect (“condition*visit”) was observed. The results suggested that for each postoperative study visit, the total DGI score was better than the pre-operative score (Figure 6 and Table 1). Visual inspection of the graphs did confirm these findings.

**Table 1:** Demographics of included patients.

Participant	Gender	Age at		Implanted ear	Etiology
		Onset symptoms	Implantation		
SP1	Male	41	46	Right	Cogan syndrome
SP2	Male	28	47	Left	Cranial brain trauma
SP3	Male	23	35	Right	Bilateral Meniere’s disease
SP4	Male	48	64	Right	Chronic otitis media
SP5	Male	30	60	Right	Idiopathic
SP6	Male	32	36	Right	Bilateral Meniere’s disease
SP7	Female	53	58	Left	Idiopathic
SP8	Male	66	78	Right	Oto- and vestibulotoxicity
SP9	Male	18	76	Right	Oto- and vestibulotoxicity
SP10	Female	43	55	Left	Bilateral otosclerosis
SP11	Female	52	69	Right	Bilateral Meniere’s disease

**Figure 7:**
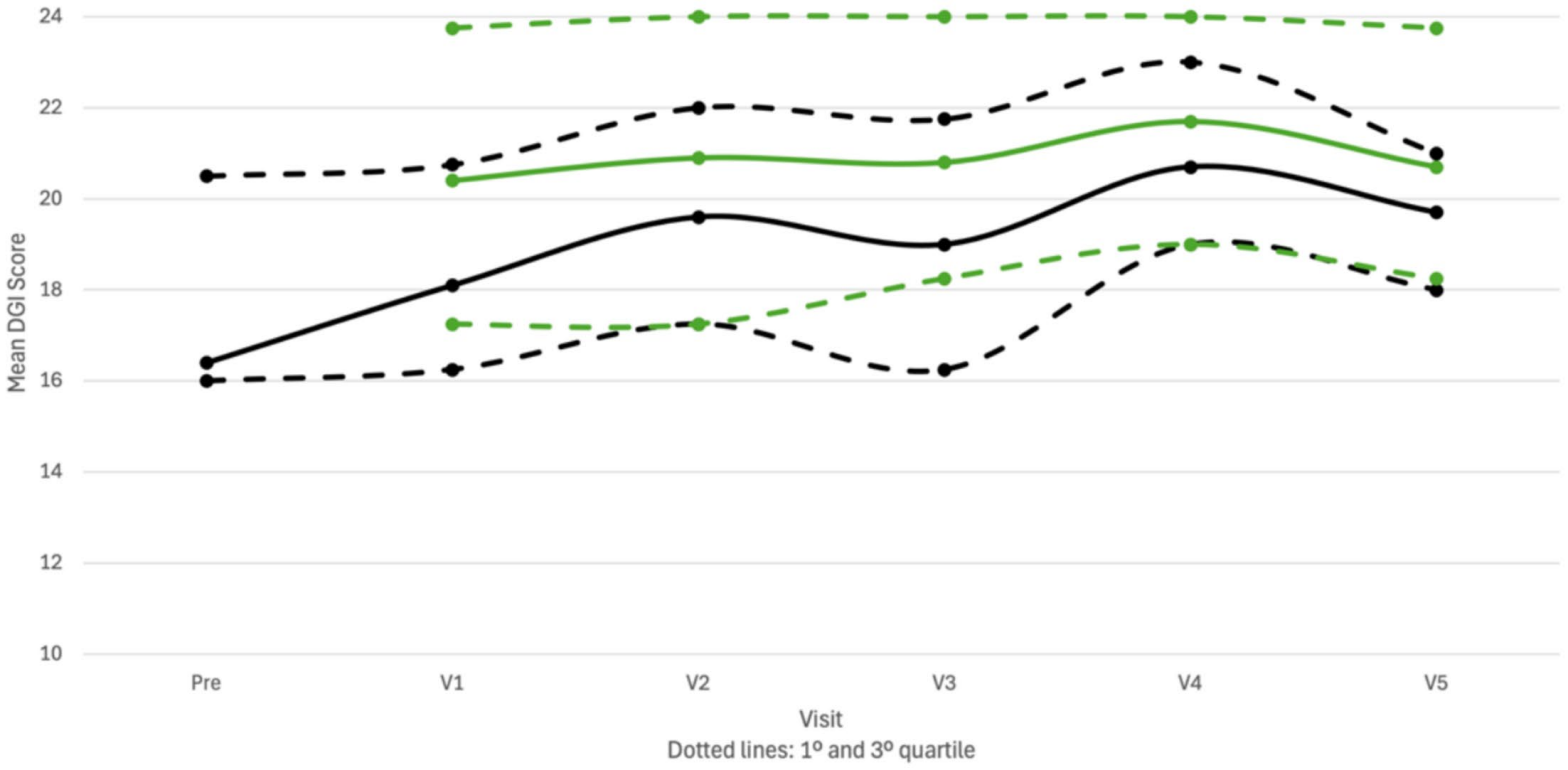
Difference in total score on the dynamic gait index (DGI) between study visits and condition of the cochleovestibular implant (CVI; ON or OFF). ON, CVI activated (green bars and full line); OFF, CVI deactivated (black bars and dotted line); pre, pre-operative data; V1-5, postoperative study visits 1 and 5 (*n* = 10).

Furthermore, a random effect was obtained [Wald *Z* = 2,244; *p* < 0.05; estimate = 8.9; standard error = 3,965; 95% confidence interval: (3.8; 21.0)] indicating that the individual DGI scores differed significantly between participants when the overall data was analyzed. By incorporating the repeated measures design (adding the “visit” term), the significance of this effect disappeared, suggesting that all participants underwent similar changes through the study visits. The total DGI score improved statistically significantly when the CVI was activated. The LMM analysis on the DGI data did show significant fixed effects regarding CVI condition (OFF vs. ON) and study visits (pre vs. postop) (Figure 7).

#### 3.3.2 Time up and go

**Figure 8:**
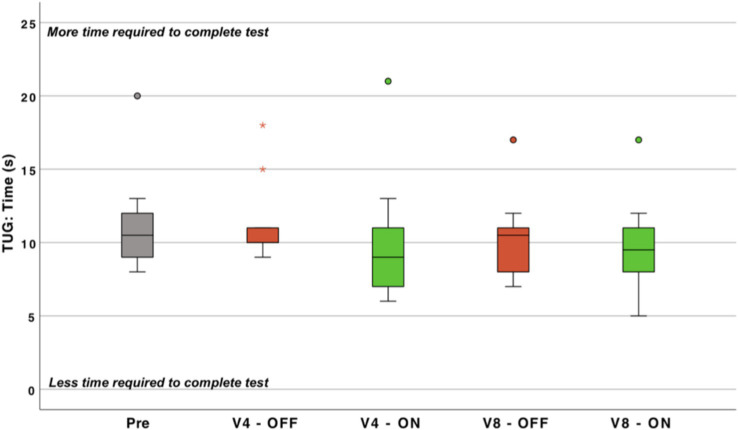
Time (s) required for performing the timed-up-and-go test (TUG) (the less time required to complete the test, the better the performance). ON, cochleovestibular implant activated; OFF, cochleovestibular implant deactivated; pre, pre-operative data; V1-5, postoperative study visits 1 to 5; Circles, first order outlier; asterisk, second order outlier (*n* = 10).

The time to complete the TUG test did not change statistically significant (*n* = 10) (Figure 8). Not reaching statistically significance is likely because of the observed ceiling effect, as 5 out of the 10 patients were in normal range before implantation.

#### 3.3.3 Posturography

In all cases the posturography was improved. The electrical stimulation of the otolithic organ, specifically the saccule, has shown significant potential to enhance stability scores in posturography assessments, such as the Sensory Organization Test (SOT). In patients with bilateral vestibulopathy, activation of the implant led to improvements across all SOT conditions.

**Figure 9:**
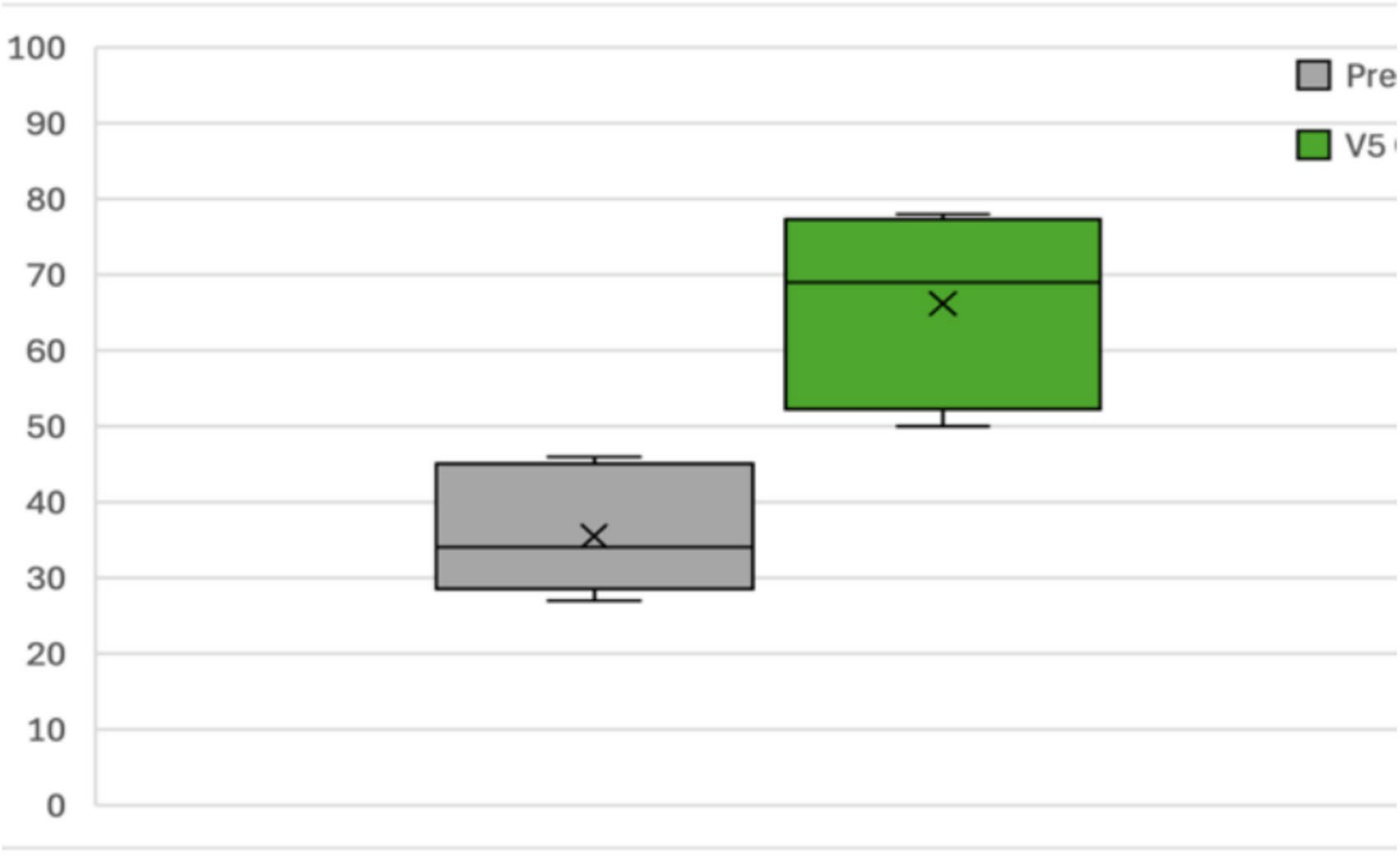
Sensory organization test results (*n* = 10).

Overall, the average preoperative global SOT score was 33%. By the final follow-up, with the implant activated, this score had increased to 68%, indicating a substantial improvement in postural stability (*p* < 0.05) (Figure 9).

#### 3.3.4 Video head impulse test

VHIT analysis was performed before and after the intervention. In all subjects and in all visits, no differences were found in the six canals results (as expected). In BVL patients, VOR gains were in all cases <0.6, before and after surgery, and no semicircular canal stimulation was intended.

#### 3.3.5 Dizziness handicap inventory

The DHI results revealed that participants perceived their disability as “moderate” or worse before implantation. The preoperative score was significantly higher than the score obtained after implant activation (Pre vs. V4 - ON: *n* = 10; *p* < 0.05; mean difference = −19.6 points, indicating improvement from the preoperative phase).

Additionally, the final postoperative measurement (V5) showed a statistically significant improvement compared to the initial postoperative measurement (V1 - OFF vs. V5: *n* = 10; *p* = 0.017; mean difference = −16.6 points, indicating further improvement).

**Figure 10:**
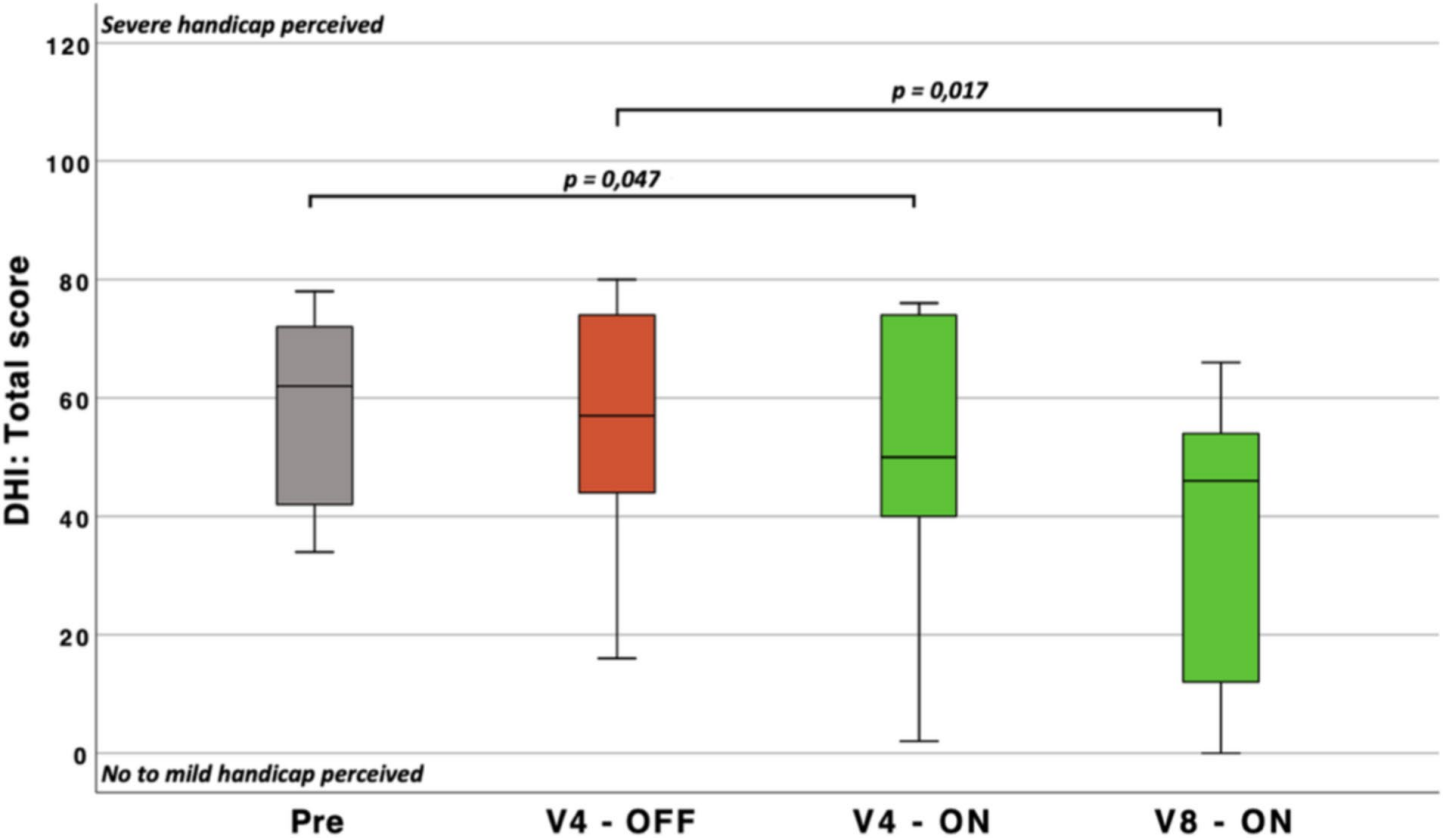
Boxplots representing the total score of the Dizziness Handicap Inventory (DHI) (the higher the score, the higher the perceived handicap mediated by the vestibular disorder). Pre, Pre-operatively; V1-5, study visits at 1 month and 9 months (respectively) after the implantation; p, probability value.

No differences were observed between the total preoperative score and the total score 1 month after implantation (V1 - OFF: prior to CVI activation) (Figure 10).

The emotional scale scores were initially statistically significant [*n* = 10; *p* < 0.05; 2(3) = 10.582], although post-hoc pairwise analyses could not confirm between which visits the difference occurred.

No statistically significant differences were observed on the physical scale.

However, the functional scale score was statistically significant lower at V5 (mean score = 17) compared to the baseline measurement at V1 (V1 - OFF: mean score = 25; *n* = 10; *p* < 0.05).

### 3.4 After effect experiment

During this clinical trial, it became clear that, although the balance of several participants improved significantly with respect to pre-op, the postoperative results obtained directly after switching off the CVI (OFF) were not that different from those obtained with CVI stimulation (ON) (cf. results section). Therefore, the team of investigators in Las Palmas performed an additional experiment to evaluate whether the CVI stimulation mediated a so-called after effect of continued improved balance after deactivation of the CVI stimulation. For this experiment, seven participants were asked to perform the TUG and DGI twice with the CVI deactivated (OFF) and once with CVI activated (ON). The OFF measurements were performed immediately after deactivation of the CVI and 24 h later (thus after 24 h of not being stimulated electrically, CVI completely OFF).

**Figure 11:**
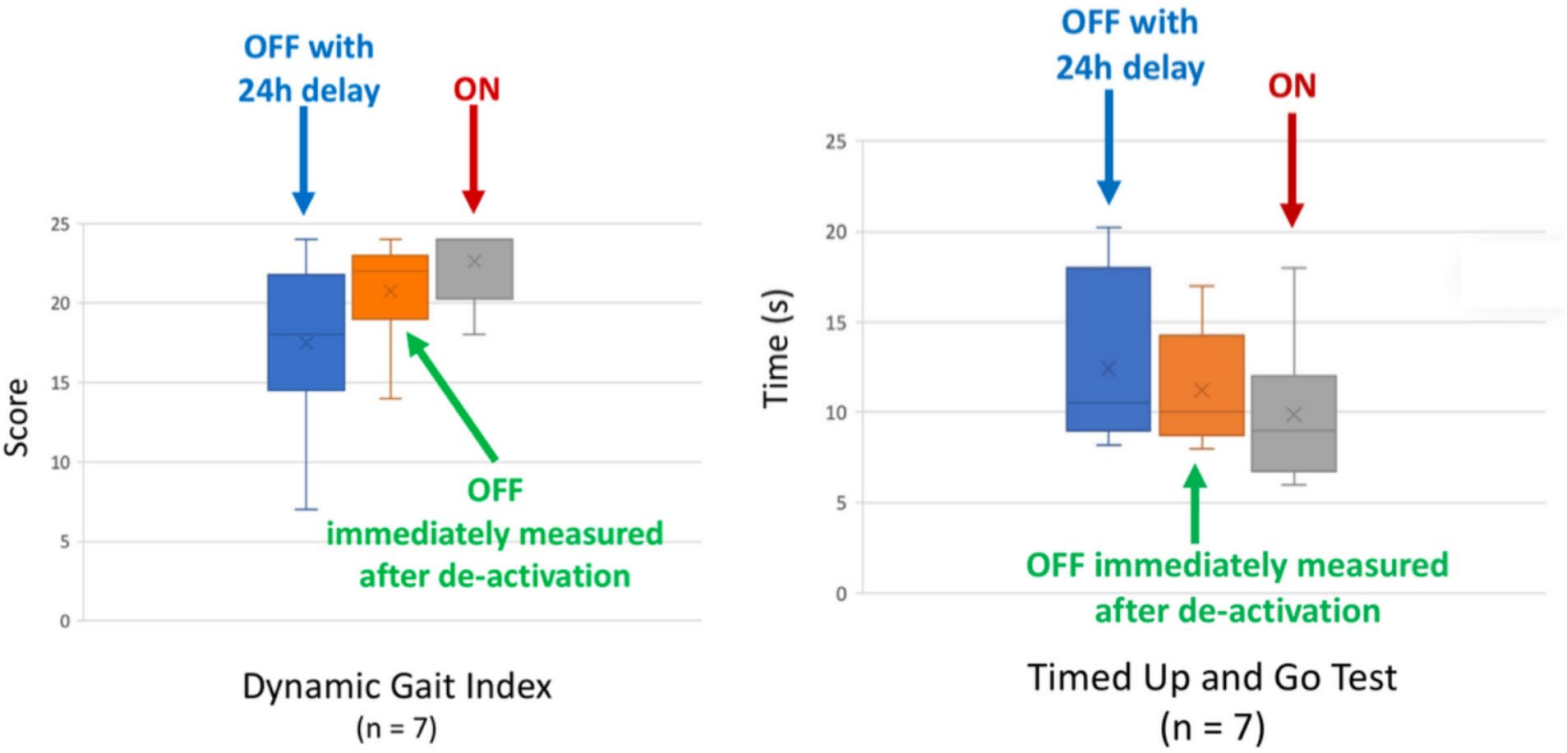
After effect and the total score on (Left) Dynamic Gait Index. The higher the DGI total score, the better the performance. ON, cochleovestibular implant activated; OFF, cochleovestibular implant deactivated. (Right) Timed Up and Go test. The shorter the time (s) to complete this test, the better. ON, cochleovestibular implant activated; OFF, cochleovestibular implant deactivated (*n* = 7).

The total DGI score during the OFF condition (measured immediately after ceasing CVI stimulation) was slightly lower (Figure 11: orange boxplot) than the total DGI score during the ON condition (Figure 11: gray boxplot). After 24 h of deactivation (i.e., receiving no CVI simulation), the participants were retested (still without CVI stimulation) (Figure 11: blue boxplot). The variability in the data increased and the mean score was lower than during the other two conditions. A similar pattern was observed during the TUG test (Figure 11).

### 3.5 Placebo effect experiment

Three stimulation maps were programmed on each participant’s sound processor. One map provided cochlear stimulation only (“CI only”) while the second map provided combined cochleovestibular stimulation (“combined CVI”). The order in which the first two maps were programmed was randomly assigned and the participants were not aware of the order. To be sure that the participants could go back to the program that they had been using the weeks and months before this experiment, they were informed that the third program was the one that they used during their daily life. The third program was thus their back-up program, which they were aware of.

The participants used programs 1 and 2 for 3 days each. After this 6day-trial, they came back to the hospital and were asked to identify programs 1 and 2 as either “CI only” or “combined CVI” based on their balance performance. If correct identification of both programs was possible, it could be assumed that the observed findings were not solely the result of a placebo effect. Additionally, datalogs were analyzed to confirm the proper use of the maps during the trial.

After the test, all patients correctly identified the “CVI” map. According to the datalog, the “CI only” map was used very minimally, while the backup map was used for the same amount of time as the “CVI” map. Therefore, based on the patients’ feedback, it was clear that they could identify which map provided vestibular stimulation. When using the “CI only” map, they perceived no vestibular benefit and consequently switched to the backup map.

## 4 Discussion

This study aimed to evaluate whether electrical stimulation of the vestibular system, specifically the inferior vestibular nerve and saccule, could improve balance in patients with severe to profound hearing loss and BVP. The findings suggest that vestibular stimulation via the CVI implant has significant potential to enhance both audiovestibular function and quality of life for this patient population.

The improvements in auditory and vestibular function were promising. All participants demonstrated better pure-tone audiometry (PTA) scores in the free-field condition and increased speech perception, often exceeding typical post-lingually implanted patient outcomes. The observed speech perception improvements during combined cochlear-vestibular implant (CVI) stimulation align with, and in some cases surpass, results from standard cochlear implant users. These gains underscore the CVI’s effectiveness in integrating both auditory and vestibular functions in daily activities.

Significant enhancements in balance were observed, particularly in dynamic gait assessments like the Dynamic Gait Index (DGI), supporting the CVI’s ability to improve functional mobility. Although improvements in static balance tests like posturography were noted, the small sample size limited statistical significance for these measures. Such findings resonate with previous studies that reported gait improvements upon semicircular canal (SCC) stimulation, suggesting that otolithic stimulation has complementary potential in restoring balance function.

Our findings align with previous research demonstrating the effectiveness of vestibular implants in restoring vestibular responses, including VEMPs (16). Earlier studies have shown that vestibular compound action potentials (ECAPs) can be elicited through electrical stimulation of the otolith organs using a paradigm similar to that of cochlear implants. These results exhibit characteristics consistent with cochlear ECAPs, supporting the validity of vestibular response telemetry (VRT) technology in measuring vestibular-origin ECAPs.

Moreover, a correlation between VRT responses and eoVEMPs has been demonstrated when the electrode is precisely placed in the otolith organs. This emphasizes the importance of stable and accurate electrode placement, as even minimal displacements can lead to significant changes in response amplitude. Notably, these responses are absent when the electrodes are incorrectly positioned or when auditory neural telemetry from cochlear implants is performed, confirming that the ECAPs recorded are vestibular rather than auditory in origin.

The ability to record such responses in 83.3% of tested electrodes highlights the potential of vestibular implants to provide effective chronic stimulation of the otolith organ in patients with bilateral vestibular dysfunction and profound sensorineural hearing loss. Additionally, studies by Perez Fornos et al. (17) have shown that vestibular rehabilitation through these devices not only partially restores the vestibulo-ocular reflex (VOR) but also improves posture and head motion stabilization, suggesting further integrative benefits for head orientation and balance.

Several hypotheses may account for these improvements. First, otolithic and semicircular canal (SCC) hair cells differ in their polarity and sensitivity to gravitational cues. Otolithic stimulation may provide a consistent reference signal for gravitational orientation, supporting the “gravitational reference” hypothesis. This reference, similar to proprioceptive feedback when stabilizing with a handhold, could help reestablish a basic sense of vertical orientation and enhance balance.

Additionally, unlike unilateral SCC stimulation, otolithic stimulation is less likely to cause tonic asymmetry, as evidenced by the absence of vertigo or dizziness reported with CVI activation. We propose that continuous electrical stimulation may substitute for the missing saccular input to the vestibular nuclei and cerebellum in patients with bilateral vestibulopathy. This input may also indirectly influence other structures involved in motor control, which have garnered significant interest recently.

One potential target is the anterior midline cerebellum (uvula), identified as a promising site for deep brain stimulation in humans to improve postural stability and gait. The midline cerebellum projects to basal ganglia structures, including the striatum, which play a critical role in gait initiation.

A second hypothesis involves the suppression of “vestibular tinnitus,” a theoretical maladaptive plasticity akin to auditory tinnitus, where chronic vestibular symptoms arise due to a lack of vestibular input. Electrical stimulation might inhibit this maladaptive neural reorganization, alleviating imbalance and chronic vestibular symptoms.This is further demonstrated in research on electrical stimulation systems for tinnitus suppression. Studies indicate that electrical stimulation of the inner ear can suppress the effects of tinnitus and, in some cases, the suppression effect persists even after the stimulation has concluded (18, 19).

The concept of stochastic resonance (SR) offers a third explanation. Low-level noise from electrical stimulation could exceed the neural activation threshold of residual vestibular function, amplifying weak vestibular responses and thus enhancing overall balance. The bell-shaped response pattern in one participant suggests SR dynamics, where excessive “noise” overstimulation impeded balance, yet optimal levels might improve it.

Additionally, CVI stimulation may activate motor control areas in the brain, particularly the anterior cerebellum, which projects to basal ganglia structures responsible for initiating gait (20, 21). This function parallels deep brain stimulation in Parkinson’s treatment, potentially aiding mobility in patients with BVP.

**Figure 12:**
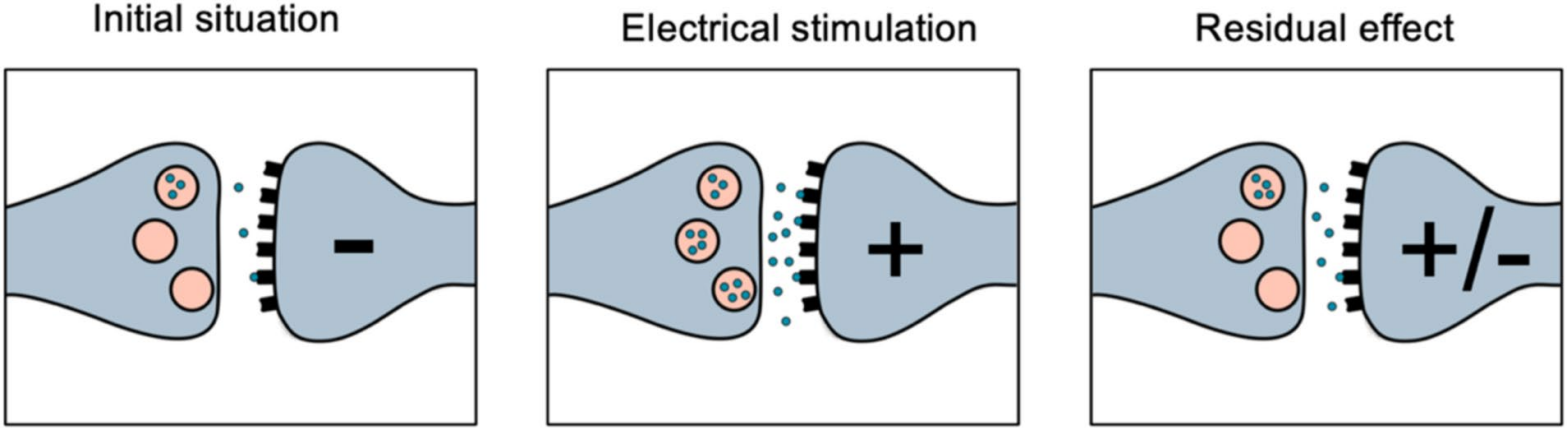
This schematics shows a dopamine synapsis and after effect phenomenon.

Building on the residual effect observed following stimulation and the potential activation of basal ganglia regions, the after-effect phenomenon could be explained as follows: Initially, the basal ganglia have a low concentration of neurotransmitters, such as dopamine. During stimulation, these ganglia are activated, promoting dopamine release. When the stimulation ceases, although dopamine generation is no longer actively promoted, residual dopamine remains present in the surrounding environment. This residual dopamine sustains the effect until it is fully reabsorbed (Figure 12).

Variability among participants suggests that factors such as residual vestibular function, etiology of BVP, and coping mechanisms could impact outcomes. This variability, coupled with possible residual or adaptive neural pathways, highlights the need for individualized parameter settings and possibly refined assessments to optimize patient outcomes. Furthermore, the observed placebo effect suggests that sensory awareness of stimulation contributes to patient performance, albeit with limitations due to non-blinded CI-only comparisons. Future studies could address this through subthreshold or placebo vestibular stimulation programs to provide more rigorous blinded comparisons.

However, several challenges and limitations persist in the development of these implants. Participant sample size on otolith implants have involved a limited number of participants due to the restrictions on inclusion criteria. Larger studies are needed to confirm the long-term efficacy and safety of otolith implants. It is also crucial to address the preservation of residual hearing in patients with partial or no hearing loss, and techniques and implant designs are in development to meet this need.

Another limitation was the after-effect seen when CVI was turned off, which likely skewed post-stimulation data. Establishing protocols to minimize or control for these effects, especially for dynamic activities like swimming, will be essential for real-world CVI applications.

## 5 Conclusion

This study demonstrates that otolith implants represent a promising technology for the treatment of BVP, a condition that severely impacts patients’ balance and quality of life.

Stimulation of the inferior vestibular nerve using otolith implants has shown significant improvements in static posture and dynamic gait in patients with BVP and severe to profound hearing loss. Furthermore, electrical stimulation of the otolithic organ can evoke action potentials in humans, providing objective evidence of neural activation in the region of the maculae.

The otolithic system plays a fundamental role in detecting all types of movement, not just linear accelerations. This robustness, along with its ability to inhibit other signals, makes it an ideal target for vestibular implants.

Chronic otolithic stimulation in humans have shown promising results, improving spatial orientation, postural stability, and gait in patients with bilateral vestibulopathy.

Otolith implants offer a promising future for treating BVP. Ongoing research focuses on optimizing stimulation parameters, exploring new paradigms and electrode designs to enhance selectivity and efficacy while minimizing crosstalk and side effects. A better understanding of the neural mechanisms is essential, particularly regarding how electrical stimulation of the otolithic organ influences neuronal activity in the central vestibular system and integrates this information to improve balance.

## Data Availability

The datasets presented in this study can be found in online repositories. The data can be found at http://www.bionicvest.eu.
